# Intronic position +9 and −9 are potentially splicing sites boundary from intronic variants analysis of whole exome sequencing data

**DOI:** 10.1186/s12920-023-01542-7

**Published:** 2023-06-26

**Authors:** Li Zhang, Minna Shen, Xianhong Shu, Jingmin Zhou, Jing Ding, Chunjiu Zhong, Baishen Pan, Beili Wang, Chunyan Zhang, Wei Guo

**Affiliations:** 1 Department of Laboratory Medicine, Zhongshan Hospital, Fudan University, Shanghai, China; 2 Department of Echocardiography, Zhongshan Hospital, Shanghai Institute of Cardiovascular Diseases, Shanghai Institute of Medical Imaging, Fudan University, Shanghai, China; 3 Department of Cardiology Shanghai Institute of Cardiovascular Diseases, Zhongshan Hospital, Fudan University, Shanghai, China; 4 Department of Neurology, Zhongshan Hospital, Fudan University, Shanghai, China; 5 Department of Neurology, Zhongshan Hospital, State Key Laboratory, Fudan University, Shanghai, China; 6 Department of Laboratory Medicine, Xiamen Branch, Zhongshan Hospital, Fudan University, Xiamen, China

**Keywords:** Intronic position +9 and −9, Intronic variants, Whole exome sequencing, WES, Intron characterization, Genetic diagnosis, Next-generation sequencing (NGS), Intronic regions flanking exons

## Abstract

Whole exome sequencing (WES) can also detect some intronic variants, which may affect splicing and gene expression, but how to use these intronic variants, and the characteristics about them has not been reported. This study aims to reveal the characteristics of intronic variant in WES data, to further improve the clinical diagnostic value of WES. A total of 269 WES data was analyzed, 688,778 raw variants were called, among these 367,469 intronic variants were in intronic regions flanking exons which was upstream/downstream region of the exon (default is 200 bps). Contrary to expectation, the number of intronic variants with quality control (QC) passed was the lowest at the +2 and −2 positions but not at the +1 and −1 positions. The plausible explanation was that the former had the worst effect on trans-splicing, whereas the latter did not completely abolish splicing. And surprisingly, the number of intronic variants that passed QC was the highest at the +9 and −9 positions, indicating a potential splicing site boundary. The proportion of variants which could not pass QC filtering (false variants) in the intronic regions flanking exons generally accord with “S”-shaped curve. At +5 and −5 positions, the number of variants predicted damaging by software was most. This was also the position at which many pathogenic variants had been reported in recent years. Our study revealed the characteristics of intronic variant in WES data for the first time, we found the +9 and −9 positions might be a potentially splicing sites boundary and +5 and −5 positions were potentially important sites affecting splicing or gene expression, the +2 and −2 positions seem more important splicing site than +1 and −1 positions, and we found variants in intronic regions flanking exons over ± 50 bps may be unreliable. This result can help researchers find more useful variants and demonstrate that WES data is valuable for intronic variants analysis.

**Supplementary Information:**

The online version contains supplementary material available at 10.1186/s12920-023-01542-7.

## Introduction

The exome has been defined traditionally as the sequence encompassing all exons of protein coding genes in the genome, it covers 1–2% regions of the genome. The method of sequencing all the exons is known as whole exome sequencing (WES) [1]. With the development of sequencing technology, WES has been more and more widely applied to clinical practice and various scientific research. It is thought to be an efficient genetic disease diagnosis method to help researchers find possible disease-causing variants, because of most disease-causing variants are in exonic regions. And WES does also work in finding novel exonic disease-causing variants or genes [2].

Exonic variants are often emphasized in WES data, however, intronic variants had been found to affect gene activity and protein production, leading to genetic disorders. Intronic variants mainly regulate biological activities by dysregulating mRNA splicing [3–7]. For instance, over 25,000 disease-causing intronic variants in the Human Gene Mutation Database (HGMD) have been reported impact splicing, and most of these pathogenic variants are located nearby the splice-junction boundaries [8]. Variants located more than 100bps away from exon would lead to pseudo-exon inclusion most commonly, because of changes in splicing regulatory elements or activation of non-canonical splice sites. Additionally, deep intronic variants can disrupt transcription regulatory motifs and non-coding RNA genes [9]. These variants can also result in either retention of the intron, complete skipping of the exon, or the introduction of a new splice site within an exon or intron. Some variants that do not disrupt or create a splice site consistent with the proposal that introns contain splicing inhibitory sequences, can activate pre-existing pseudo splice sites. Some variants alternatively spliced exons and in consequence cause disease, can affect the fine balance of isoforms produced [10]. However, the characterization of these intronic variants in WES data is still unknown.

In this study, we analyzed 269 whole exome sequencing data to describe characteristics of intronic variants in data from conventional WES testing, which include: (1) the number of intronic variants in the WES data and whether the variant count of different positions in intronic regions flanking exons which are defined as the region upstream/downstream of the exon (default is 200 bps) have significant difference. (2) The proportion of false variants which cannot pass quality control (QC) and the number of pass variants which can pass QC at the different intron position, and whether these proportion and variants number between different position have significant different. (3) Whether the false proportion between intronic variants of flanking regions and exonic variants have significant different. (4) The number of deleterious variants which are predicted to be damaging by prediction software and the deleterious variants proportion of pass variants, and whether the deleterious variants number and proportion at different position of flanking regions have significant different. We hope that the result can help researchers better understand the characteristics of intronic variants in conventional WES testing data and find some meaningful intronic variants for genetic disease diagnosis or research study, finally improve the clinical diagnostic value of WES.

## Method

### Analysis of total raw variants called from WES data

**Fig. 1:**
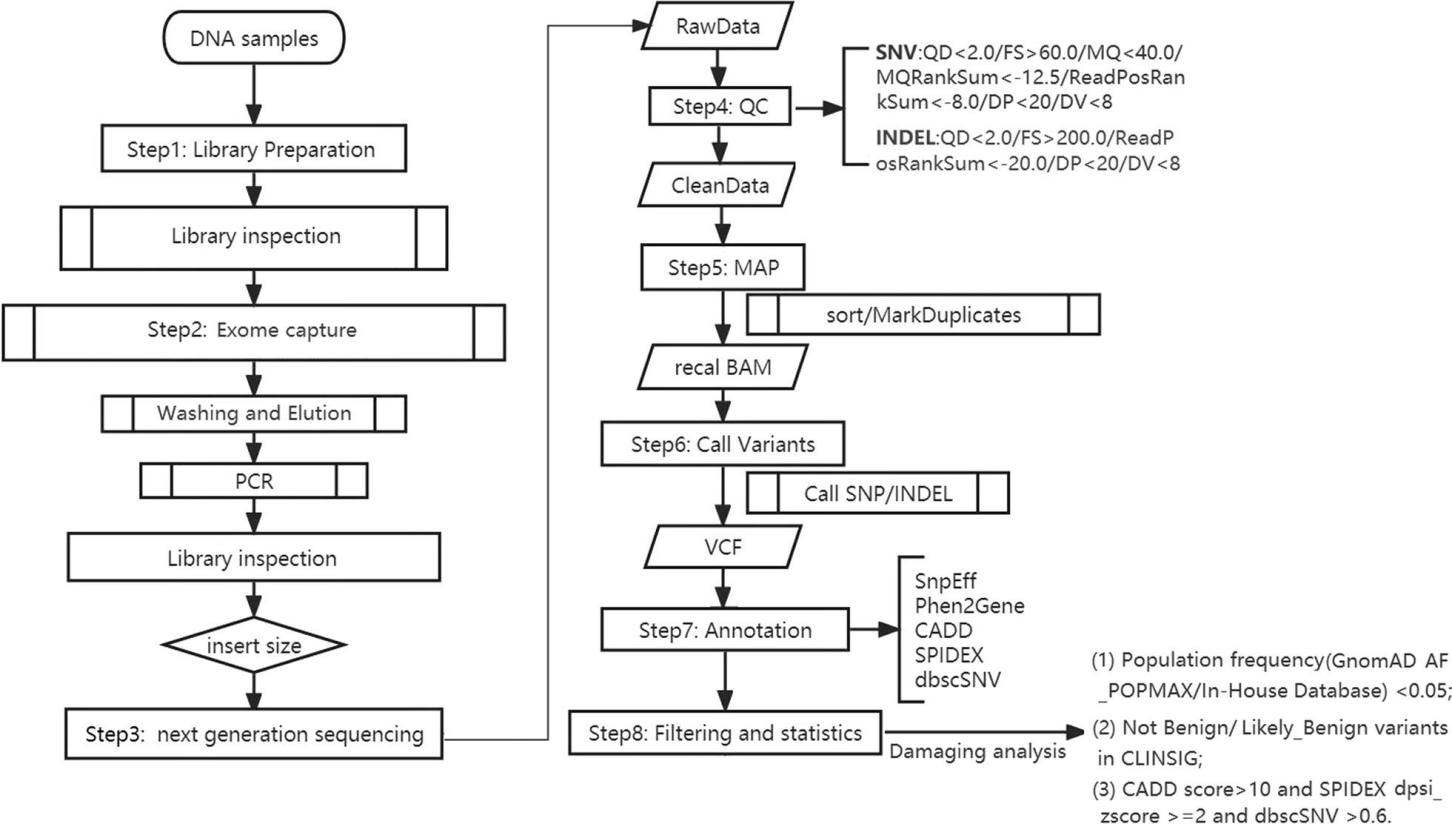
Experimental procedure flowchart. The left section depicted the WES workflow, whereas the work low for quality control, variant filtering and bioinformatics analyses was illustrated in the right section

The 269 WES data was from our WES genetic testing project for patients with adult genetic disorders, including cardiovascular diseases, nervous system diseases, digestive disease, endocrine disease, reproductive system disease, etc. (Table 1). Patients with cardiovascular and nervous system diseases made up most of the population. The exome capture kit used in the current experiment was Exome Plus Panel V2.0 provided by the medical laboratory of Nantong ZhongKe Co. Ltd (Nantong 226000, China). This kit spanned a 46.7 Mb target exome region of the human genome. The bioinformatics pipeline was as bellow: To ensure the reliability of the results, there were a series of QC steps for variants calling. Fastp (https://github.com/OpenGene/fastp) [11] and self-developed software were used to filter raw sequencing data. The adapter and the reads which were too short would be removed. When the number of N (N means that the base information could be determined) in the paired-end reads was longer than 5 bp, these reads needed to be removed. When the proportion of low-quality bases (base quality scores less than 20) contained in the paired-end reads more than 40%, these reads also needed to be removed. The sequencing data filtered by above QC steps was called CleanData, it could be used for next alignment step. The alignment softwares included bwa-mem2 (https://github.com/bwa-mem2/bwa-mem2) [12], samtools (https://github.com/samtools/samtools) [13] and sambamba (https://github.com/biod/sambamba) [14]. Bwa-mem2 was selected to align CleanData to the reference genome (hg19) and generate SAM files, samtools was selected to sort SAM files according to chromosome positions and converted them into BAM files, sambamba was selected to mark duplication reads generated from PCR amplification in the BAM files. These BAM files would be used for variant calling. SNV (Single Nucleotide Variant) and InDel (Insertion or Deletion) variant calling software was GATK (https://github.com/broadinstitute/gatk/releases) [15], the HaplotypeCaller module was used to call variant from BAM files. The total variants called from 269 WES data were called Raw variants. Then the variants would be annotated, the major annotation softwares and in silico predictive algorithms for each variants were snpEff (http://pcingola.github.io/SnpEff/) [16], Annovar (https://annovar.openbioinformatics.org/) [17], Phen2Gene (https://github.com/WGLab/Phen2Gene) [18], CADD (https://cadd.gs.washington.edu/) [19], SPIDEX (https://www.openbioinformatics.org/annovar/spidex_download_form.php) [20], dbscSNV (http://www.liulab.science/dbscsnv.html) [21], and self-developed software. The major annotation database included Clinvar, 1000 Genomes, gnomAD, dbSNP, OMIM, and in house database (Fig. 1).

**Table 1:** Clinical characteristics of patients

Disease of patients	Age (years)	No. of male/female	No. of patients
Cardiovascular diseases	22–58	77/56	133
Nervous system diseases	23–56	43/33	76
Rheumatic disease	22–44	9/7	16
Reproductive system disease	25–50	6/8	14
Endocrine disease	26–58	5/5	10
Digestive disease	22–52	7/2	9
Kidney disease	23–30	2/4	6
Respiratory diseases	22–49	2/3	5

### Analysis of false variants

Raw variants were filtered by HardFilter module, and the QC filter parameters included (1) SNV: QD < 2.0 or FS > 60.0 or MQ < 40.0 or MQRankSum < − 12.5 or ReadPosRankSum < − 8.0 or DP < 20 or DV < 8; (2) INDEL: QD < 2.0 or FS > 200.0 or ReadPosRankSum < − 20.0 or DP < 20 or DV < 8. These QC pass variants were called Pass variants. Variants which could not pass QC filtering would be counted as False variants, which might be caused by sequencing errors and PCR amplification. The proportion of false variants were called FP. To find the relationship between the FP and the sequencing depth of different positions, we calculated the average sequencing depth for each position of intronic region (Fig. 1).

### Analysis of damaging intronic variants

The damaging analysis focused on the intronic regions flanking exons which was upstream/downstream region of the exon (default is 200 bps), and the damaging filter parameters included (1) population frequency (GnomAD_AF_POPMAX/In-House Database) < 0.05; (2) Not Benign/Likely_Benign variants in CLINSIG; (3) CADD score > 10 and SPIDEX dpsi_zscore > = 2 and dbscSNV > 0.6. These variants were called Deleterious variants (Fig. 1).

### Analysis of statistical differences between each group above

T-test and Fisher's exact test were used to determine if there was a significant difference between the observed groups which include: (1) If the raw variants number of different positions in intronic regions flanking exons had significant difference. (2) If the FP of different positions in intronic regions flanking exons had significant difference (3) If the pass variants number of different positions in intronic regions flanking exons had significant difference. (4) If the deleterious variants number of different positions in intronic regions flanking exons had significant difference. (5) If the FP between variants in intronic regions flanking exons and variants in exonic region had significant different. When the p-value was 0.05 or lower, the result was trumpeted as significant, but if it was higher than 0.05, the result was non-significant.

## Results

### The number of raw/pass/deleterious variants and the number of genes associated with these variants

**Fig. 2:**
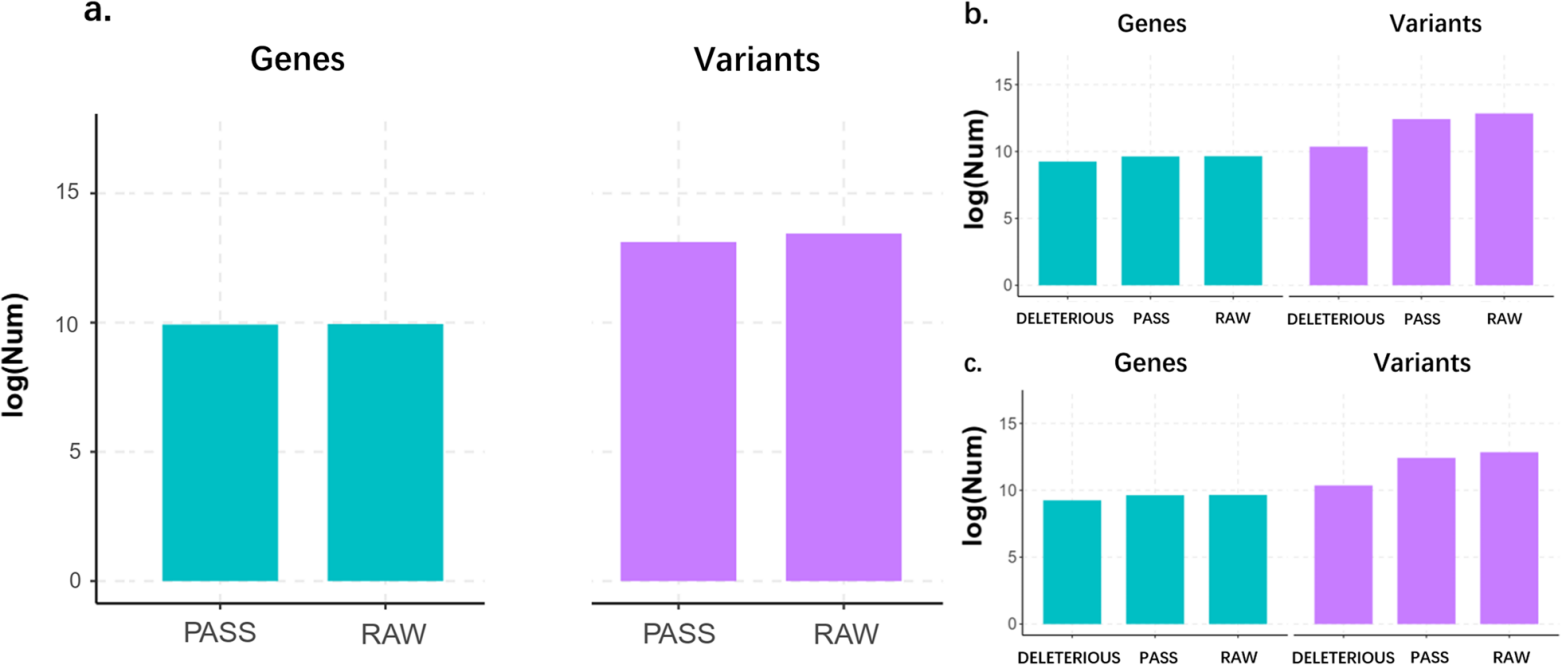
Number of total variants and its associated genes of the 269 WES data. **a** Number of total raw variants and pass variants and genes associated with these variants. There were 688,778 unique raw variants and 496,711 pass variants called from 269 WES data, genes associated with these variants were 20,791 and 20,366. **b** Number of raw, pass and deleterious intronic variants and genes associated with these variants. There were 377,807 raw intronic variants, 246,656 pass intronic variants and 31,473 deleterious intronic variants, genes associated with these variants were 15,531, 15,128 and 10,325. **c** Number of raw, pass and deleterious intronic variants in intronic regions flanking exons and genes associated with these variants. There were 367,469 raw variants, 242,142 pass variants and 31,473 deleterious variants in intronic regions flanking exons, genes associated with these variants were 15,449, 15,052 and 10,325

The average sequencing depth of all 269 WES was more than 100X in target region and 99% region was with more than 20X sequencing depth. If the variant was detected in multiple samples, the number of this variant was still counted as 1. Finally, 688,778 unique raw variants detected from 20,791 genes were called from 269 WES data, which contain 377,807 intronic variants and 310,971 exonic variants (Additional file 1: Table S1). Among the intronic variants, 367,469 variants detected from 15,449 genes were in intronic regions flanking exons. After QC filter, there remained 496,711 pass variants detected from 20,366 genes which contained 246,656 intronic variants and 250,055 exonic variants. Among the intronic pass variants, 242,142 variants detected from 15,052 genes were in intronic regions flanking exons. After damage filter, there were 31,473 deleterious variants detected from 10,325 genes in intronic regions flanking exons (Fig. 2) (Additional file 2: Table S2).

### The distribution of the raw intronic variants number

**Fig. 3:**
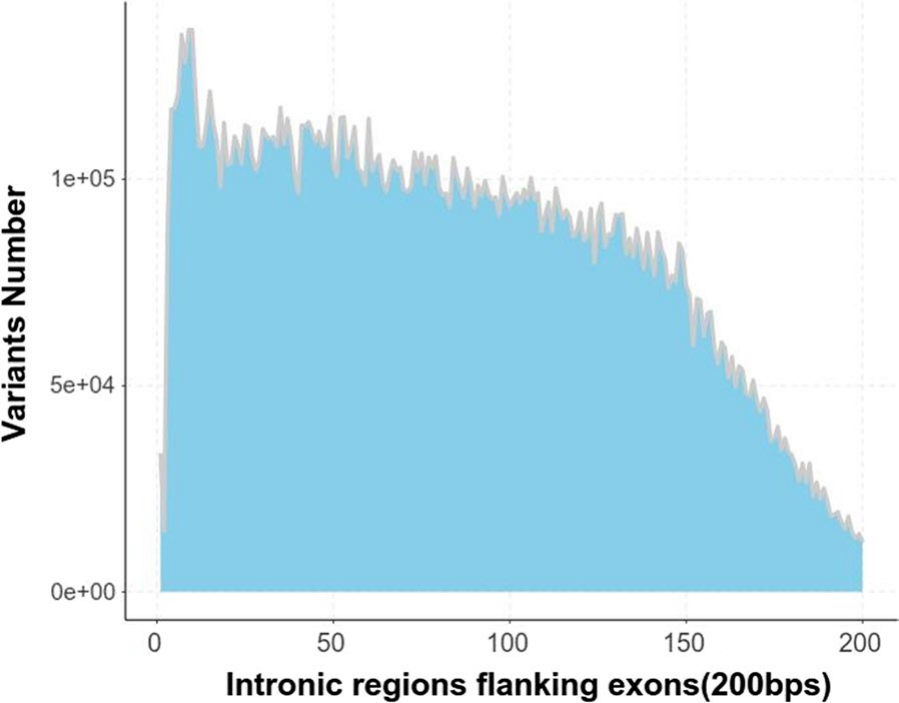
Distribution of raw intronic variants number. The variant number of the +2 and −2 positions was 14,744 which was less than the +1 and −1 positions. After the second position, these variant number gradually raised, then reached the largest number 136,241 at the +9 and −9 positions. Then the number gradually and gently decreased with ups and downs, before the +149 and −149 positions the descent slope was smaller, after that the descent slope was close to −1

The distribution of the raw variants number in intronic regions flanking exons was like below: The variant number of +2 and −2 positions was 14,744 which exceeded our expectations while being less than +1 and −1 positions, this result indicated that +2 and −2 positions might be more conserved than +1 and −1 positions in evolutionary process. The variant number of +2 and −2 positions was the fifth from bottom, the last four was 14,660, 12,941, 14,059, 12,237 from +197 and −197 positions, +198 and −198 positions, +199 and −199 positions, +200 and −200 positions. After the second position, the variant number gradually raised, then reached the largest number 136,241 at the +9 and −9 positions, which made us consider that the +9 and −9 positions might be a potential boundary sites affecting splicing or gene expression. Then the number gradually and gently decreased with ups and downs, before the +149 and −149 positions the descent slope was smaller, after that the descent slope was close to −1 (Fig. 3) (Additional file 3: Table S3).

### The FP distribution of the raw intronic variants

**Fig. 4:**
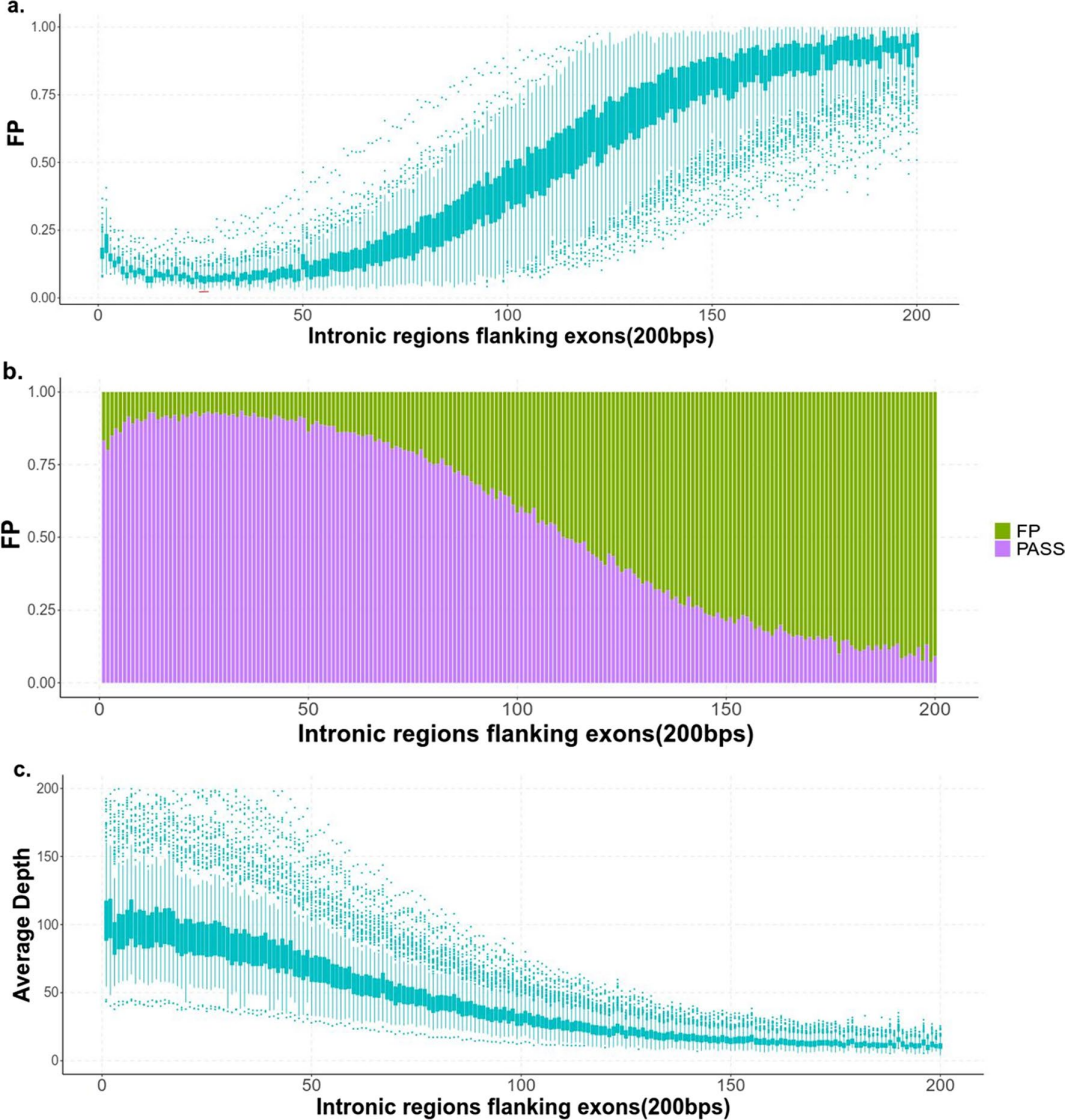
The FP distribution of the intronic variants. **a** The FP distribution of the raw intronic variants. The FP of the +2 and −2 positions was higher than the +1 and −1 positions. Then it was slowly gradually decrease until to +26 and −26 positions it transformed to gradually raise. After the +50 and −50 positions, it increased significantly until to +192 and −192 positions it tended to be stable. **b** The fill FP distribution of the raw intronic variants. The FP distribution rule of the raw intronic variants revealed from this picture was the same as that in figure a. **c.** The distribution of average depth at different positions in intronic regions flanking exons which was inversely proportional to the FP distribution

The FP distribution of the raw variants in intronic regions flanking exons was like below: The FP of the +2 and −2 positions was about 16–18%, it was higher than the +1 and −1 positions which was about 10–12%. Then FP was slowly gradually decrease, until to the +26 and −26 positions it transformed to gradually raise which was about 5–6%. After the +50 and −50 positions, it increased significantly. Then to +192 and −192 positions it tended to be stable which was about 80–90%. The distribution of FP generally accorded with “S”-shaped curve. However, the distribution of average depth at different positions in intronic regions flanking exons was inversely proportional to the FP distribution, which indicated false intronic variant in WES data might mainly result from low coverage (Fig. 4) (Additional file 4: Table S4).

### The number distribution of the pass intronic variants

**Fig. 5:**
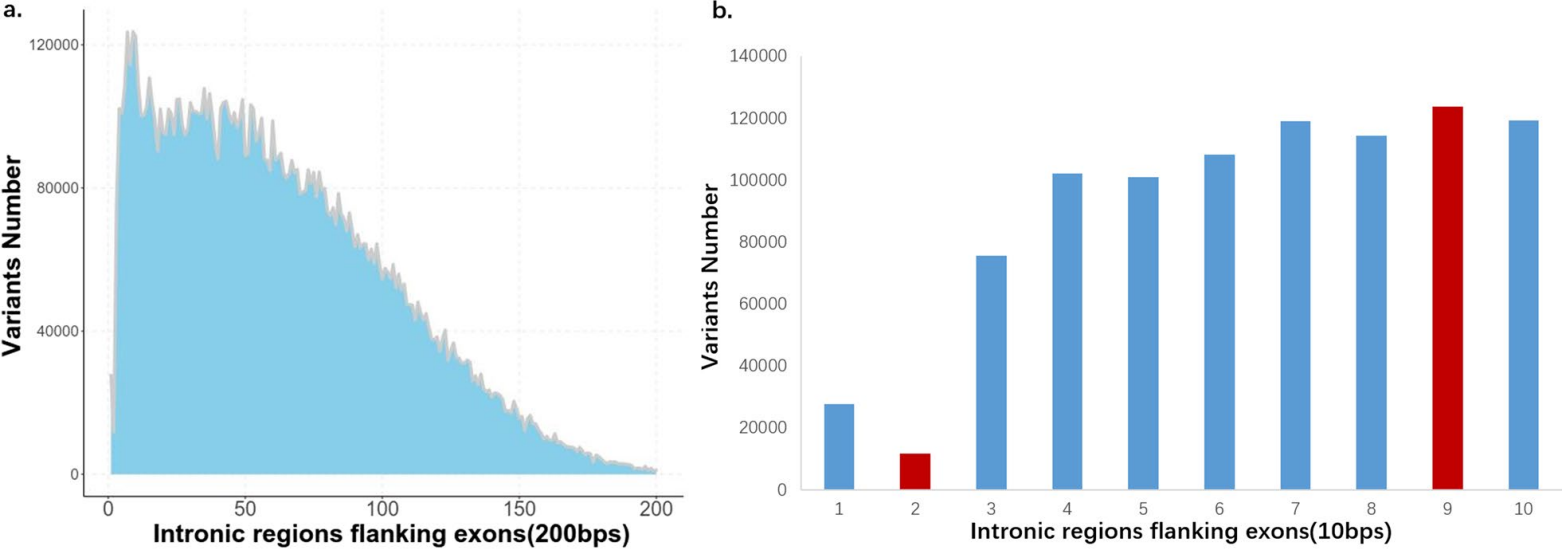
Distribution of pass intronic variants number. **a** Distribution of pass intronic variants in intronic regions flanking exons (200 bp). The least variant number in the intronic regions flanking exons within 150bps was at +2 and −2 positions which was less than the +1 and −1 positions. After the second position, these variant number gradually raised and reaching the largest number at the +9 and −9 positions. Then it gradually decreased with ups and downs. The number basically decreased with decreasing sequencing depth. **b** Distribution of pass intronic variants in intronic regions flanking exons (10 bp). The largest variant number 123,696 was at the +9 and −9 positions, and then the number gradually decreased with ups and downs

The distribution of the pass variants number was like below: Like the raw variants number, the variant number of the +2 and −2 positions was less than the +1 and −1 positions which was 11,788, and it was the least one in the intronic regions flanking exons within 150bps, the evolutionary conservatism and importance of +2 and −2 positions had been further demonstrated. After the second position, these variant number gradually raised until to the largest number 123,696 at the +9 and −9 positions, which further indicated it might be a important boundary sites affecting splicing or gene expression. Then it was gradually decreased with ups and downs, and the number basically decreased with decreasing sequencing depth (Fig. 5) (Additional file 5: Table S5).

### The number distribution of the deleterious intronic variants

**Fig. 6:**
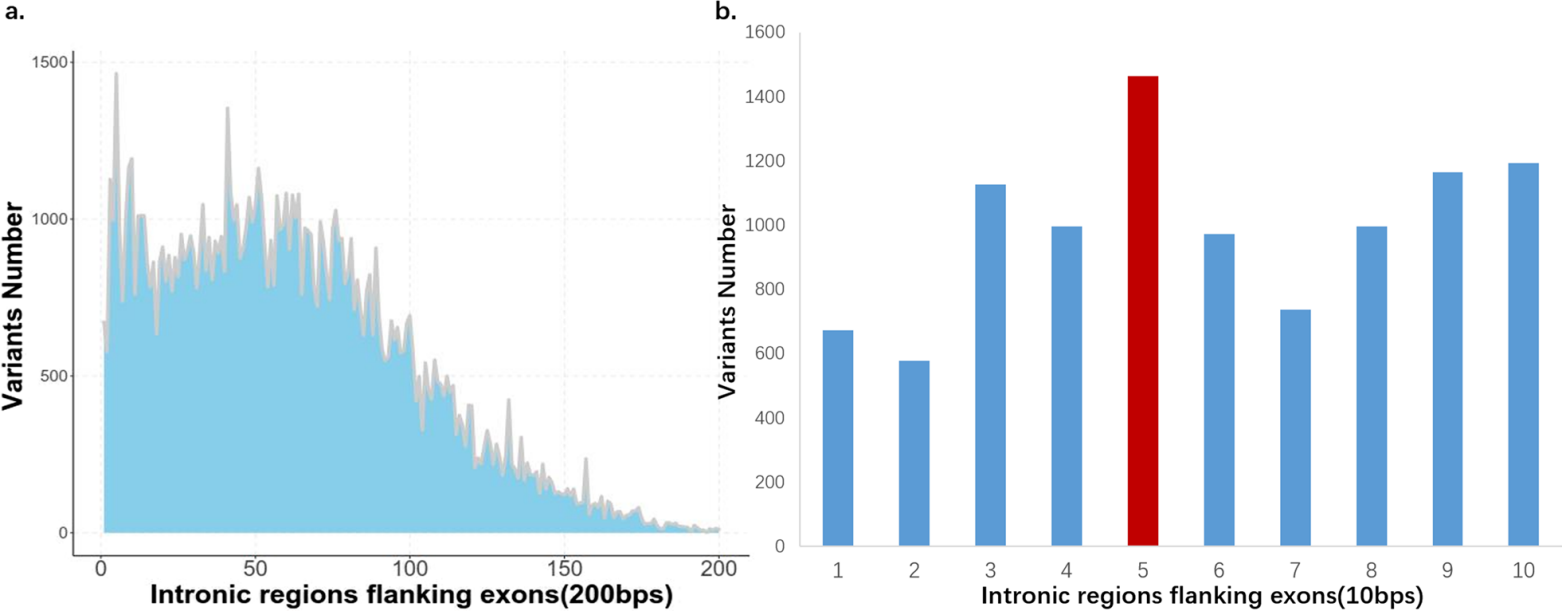
**a** Distribution of deleterious intronic variants in intronic regions flanking exons (200 bp). The largest number appears at the +5 and −5 positions, then the number dropped until at the +9 and −9 positions to +14 and −14 positions. After this the number gradually decreased with ups and downs. Then the number was gradually irregular decreasing with ups and downs. **b** Distribution of deleterious intronic variants in intronic regions flanking exons (10 bp). The largest number 1464 appeared at the +5 and −5 positions

The distribution of the deleterious variants number in intronic regions flanking exons was like below: the largest number 1464 appeared at the +5 and −5 positions, which suggested that +5 and −5 positions might be a potential pathogenic hot spot. Then the number dropped until at the +9 and −9 positions to +14 and −14 positions which was 1011 to 1165. After this the number gradually decreased with ups and downs. At several positions, the variant number was relatively large. Such as it was 1046 to 1354 between the +40 and −40 positions and +44 and −44 positions, 1045 to 1162 between the +48 and −48 positions to +52 and −52 positions, 1077 to 1080 between the +62 and −62 positions to +64 and −64 positions. Then the number was gradually irregular decreasing with ups and downs (Fig. 6) (Additional file 6: Table S6). Combined with the results of number distribution of the pass intronic variants, these results suggested that there might no direct correlation between the pass intronic variants number and the deleterious variants number at the position in intronic regions flanking.

### Statistical difference of the raw/pass/deleterious intronic variants number at different positions

**Fig. 7:**
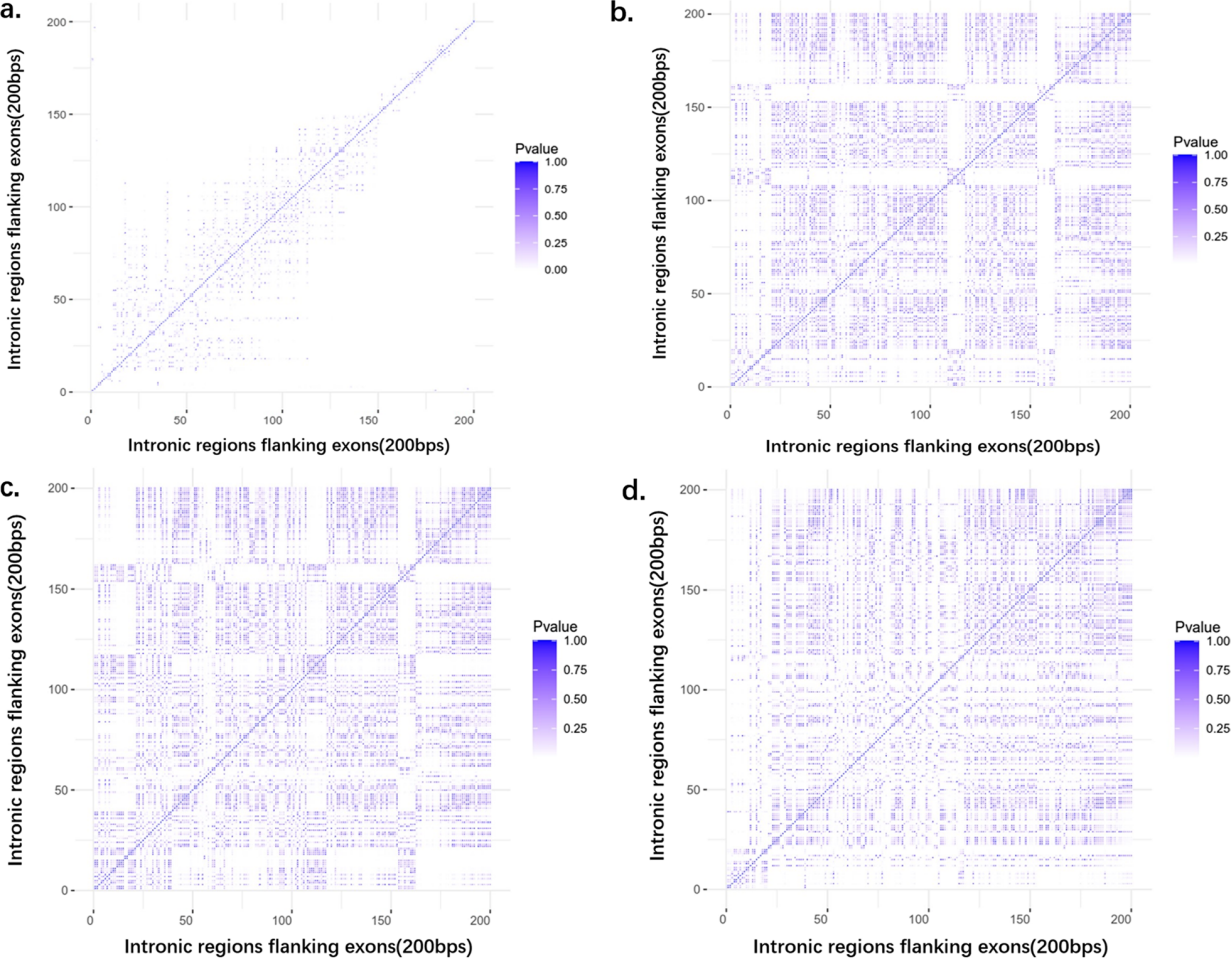
Statistical differences of the raw/pass/deleterious intronic variants number at different positions. **a** The statistical differences of raw intronic variant number at different positions. The P-value of t-test decreases with distance away from exons when the variants were at the intronic regions flanking exons between 20 and 150 bp, about half of them was significant difference (p < 0.01). The distance between which the variants number was significant difference was basically more and more small with the position away from nearby exons was more and more far. If the region were within 20 bp or more than 150 bp from nearby exon, the variants number at different position in these regions was always significant difference (p < 0.01). **b** The statistical differences of raw intronic variants FP at different positions. The proportion of false variants was non-significant difference (p > 0.05) between most positions. **c** The statistical differences of pass intronic variants number at different position. The number of pass variants was non-significant difference (p > 0.05) between most positions. **d** The statistical differences of deleterious intronic variant number at different positions. The number of deleterious variants was significant difference (p < 0.05) between some positions

The raw variants number statistical analysis results of different positions in intronic regions flanking exons were as below: The P-value of t-test decreased with distance away from exons when the variants were at the intronic regions flanking exons between 20 to 150 bp. When the distance between each position was more than 60 bp, the variants number of the different position was significant difference (p < 0.01), and this distance was more and more small with the position away from nearby exons was more and more far expect at the +18 and −18 positions, +20 and −20 positions, +40 and −40 positions and so on. If the region was within 20 bp or more than 150 bp from nearby exon, the variants number at different position in these regions was always significant difference (p < 0.01), expect when we compared the first (± 1) position with the +2 and −2 positions, +3 and −3 positions, +185 and −185 positions to +195 and −195 positions, and so on (Fig. 7a).

We compared the FP and the number of pass variants and deleterious variants at different positions in intronic regions flanking exons with each other, the FP and the number of pass variants was non-significant difference (p > 0.05) between most positions. The number of deleterious variants was significant difference (p < 0.05) between some positions, expect when we compared the position at ± 1 versus ± 2, ± 1 versus ± 4 ~ ± 5, ± 9 versus ± 3 ~ ± 8, ± 10 versus ± 1 ~ ± 2, ± 10 versus ± 4 ~ ± 7, ± 11 versus ± 4 ~ ± 10, ± 13 ~ ± 14 versus ± 4 ~ ± 11, ± 18 ~ ± 20 versus ± 4 ~ ± 11, ± 29 ~ ± 32 versus ± 23 ~ ± 26, ± 35 ~ ± 38 versus ± 23 ~ ± 26, ± 35 ~ ± 38 versus ± 30 ~ ± 32, ± 40 ~ ± 46 versus ± 23 ~ ± 26, ± 40 ~ ± 46 versus ± 29 ~ ± 32, ± 40 ~ ± 43 versus ± 23 ~ ± 26, ± 45 ~ ± 46 versus ± 40 ~ ± 44, 48 versus ± 23 ~ ± 26, ± 48 versus ± 29 ~ ± 32, ± 60 versus ± 35 ~ ± 51, ± 63 versus ± 23 ~ ± 52, ± 65 ~ ± 66 versus ± 23 ~ ± 52, ± 69 versus ± 23 ~ ± 52, ± 71 ~ ± 73 versus ± 23 ~ ± 25, ± 71 ~ ± 73 versus ± 30 ~ ± 32, ± 123 ~ ± 124 versus ± 35 ~ ± 38, ± 128 ~ ± 129 versus  ± 35 ~ ± 38, ± 123 ~ ± 128 versus ± 40 ~ ± 46, ± 123 ~ ± 128 versus ± 48, ± 122 ~ ± 128 versus ± 51 ~ ± 52, ± 123 ~ ± 128 versus ± 84 ~ ± 87, ± 123 ~ ± 128 versus ± 92 ~ ± 93, ± 129 versus ± 106 ~ ± 114, ± 123 ~ ± 128 versus ± 118 ~ ± 120, ± 127 versus ± 122 ~ ± 126, ± 128 versus ± 123 ~ ± 127, ± 131 ~ ± 143 versus  ± 23 ~ ± 26, ± 131 ~ ± 143 versus ± 29 ~ ± 32, ± 131 ~ ± 133 versus  ± 35 ~ ± 38, ± 136 ~ ±  ~ ± 139 versus ± 35 ~ ± 38, ± 131 ~ ± 135 versus ± 40 ~ ± 46, ± 137 ~ ± 138 versus ± 40 ~ ± 46, ± 140 ~ ± 143 versus ± 41 ~ ± 46, ± 131 versus ± 48 ~ ± 52, ± 134 ~ ± 135 versus ± 48 ~ ± 53, ± 140 versus ± 48 ~ ± 53, ± 143 versus ± 48 ~ ± 53, ± 131 ~ ± 135 versus  ± 65 ~ ± 66, ± 130 ~ ± 148 versus ± 63, ± 137 ~ ± 148 versus ± 65 ~ ± 66, ± 168 ~ ± 174 versus ± 106 ~ ± 114, ± 175 ~ ± 192 versus ± 118 ~ ± 120, ± 178 ~ ± 192 versus  ± 123 ~ ± 128, ± 175 versus ± 118 ~ ± 153, ± 194 ~ ± 200 versus ± 122 ~ ± 128, ± 168 ~ ± 192 versus ± 131 ~ ± 133, ± 168 ~ ± 174 versus ± 154 ~ ± 166, ± 194 ~ ± 200 versus ± 181 ~ ± 192, ± 188 ~ ± 192 versus ± 175 ~ ± 187, ± 181 ~ ± 192 versus ± 140 ~ ± 153, ± 178 versus ± 118 ~ ± 143, and so on (Fig. 7). These results suggested the raw variants number at different position in intronic regions flanking exons might always significantly different, but if the distance between each position was not far, the pass variants number at different position in intronic regions flanking exons were often not significantly different.

### Statistical differences of the FP between intronic regions flanking exons and exonic region

**Fig. 8:**
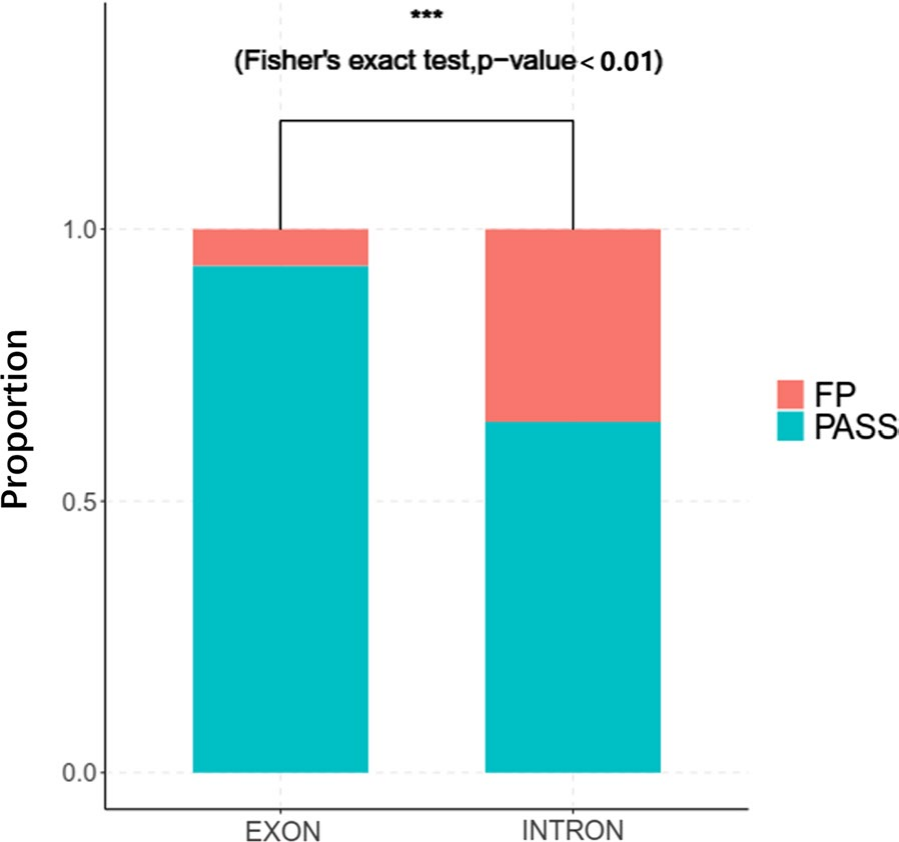
The statistical differences of FP between intronic regions flanking exons and exonic region. The FP in intronic regions flanking exons was much greater than exonic variants, the difference between these two groups was significant (p-value = 1.9228 × 10^−60^)

We compared the FP between intronic regions flanking exons and exonic region, there was significant difference (p-value = 1.9228 × 10^–60^) between these two groups, the FP in intronic regions flanking exons was much greater than exonic variants, which might result from the average sequencing depth in exonic region was much more than intronic region (Fig. 8).

## Discussion

Introns are a hallmark of eukaryotic evolution, and a substantial intron gain has accompanied the origin of metazoan [22]. Several studies have shown that intronic variant is very important for genetic diseases clinical diagnosis and sometimes as a cause of monogenic disorders and hereditary cancer syndromes [9, 23]. Hamvas et al. found that Genetic variants in intron 4 of the surfactant protein B gene *SFTPB* is associate with pulmonary morbidity in newborn infants and adults [24]. Weisschuh et al. assigned pathogenicity to *POC1B* novel deep Intronic and non-canonical splice site variants [25]. Qian et al. identified deep-intronic splice mutations in a large cohort of patients with inherited retinal diseases [26]. Li et al. raveled synonymous and deep intronic variants causing aberrant splicing in two genetically undiagnosed epilepsy families [27]. Fitzgerald et al. found that a deep Intronic variant activates a pseudo exon in the *MTM1* gene in a family with X-Linked myotubular myopathy [28]. Lin et al. found that intronic variants can impact alternative splicing by interfering with splice site recognition that 5′-splice sites of exon 20 in the *IKBKAP* gene causes skipping of exon 20, resulting in malfunction of *IKBKAP* in 99.5% of familial dysautonomia (FD) cases [29]. mRNA sequencing is a way to identify intronic splicing variants [7]. However, in the current clinical diagnosis and treatment process, to provide patients with the most cost-effective testing, WES is recommended as a first-tier test. If the result is negative and the patient’s phenotype or family history is very consistent with genetic disorders, the clinicians will advise them moving on to the next step of RNA-seq to detect the potential pathogenic splicing variants. RNA-seq has a lot of value in identifying intronic splicing variants, but if WES data can be better used to identify splicing variants, this may bring better benefits to patients. In our study, we hoped to detect pathogenic splicing variants as much as possible in the detected WES data of patients to save the need for RNA-seq detection and further improve the clinical diagnostic value of WES. However, there was no studies overall reported the clinical significance of different intronic variations in intronic regions flanking exons.

WES is more widely used than whole genome sequencing (WGS) in the clinical setting due to lower cost and more manageable data volumes. Reanalyzing WES raw data is recommended before performing WGS [30]. To improve the clinical diagnostic value of WES, 269 WES data from our WES genetic testing project of patients with adult genetic disorders were re-analysed. We revealed the characteristics of intronic variant in conventional WES testing data for the first time. The results analyzed from 269 WES data showed that, at +9 and −9 positions the number of pass intronic variants was most, at +2 and −2 positions it was least. And the number basically decreased with decreasing sequencing depth. The pass variants number of +2 and −2 positions was inconsistent with expectation. The probable reason is that the +2 and −2 positions may be a more important splicing site than +1 and −1 positions and in which variants cannot be generated arbitrarily during evolution [6]. Xu et al. [31] found that variants at +1 and −1 positions did not abolish splicing completely, but position +2 had the most severe effect on trans-splicing. The pass variants number of +9 and −9 positions was the most, this position had been reported some pathogenic variants [32, 33]. Semlow et al. [34] found that during pre-mRNA splicing a substitution of +2 and −2 to +9 and −9 with an equivalent-length carbon spacer would destabilize interactions with bound factors, permitted efficient branching. In a mutational analysis of U12-dependent splice site dinucleotides, Dietrich et al. [35] found that in the +9 and −9 mutants the usage of the upstream AG/ was only moderately reduced both in vivo and in vitro. All of these indicate that the +9 and −9 positions are potentially splicing sites boundary, we will further confirm this result through functional experiments.

We also found the farther away from the nearby exon, the less the number of pass variants. The FP in the intronic regions flanking exons generally accorded with “S”-shaped curve. However, the distribution of average depth at different positions in intronic regions flanking exons was inversely proportional to the FP distribution. As we thought, in the WES sequencing data the FP of variants in intronic regions flanking exons was much greater than in exonic regions. The false variants can be caused by low coverage, sequencing errors, and PCR amplification [36]. The sequencing and variants calling accuracy will be reduced at lower sequencing depths region and lower coverage region. Variants in these regions are always not true variants. In this study we found the farther away from the nearby exon, the less the number of pass variants, thus false intronic variant in WES data may mainly result from lower coverage and lower sequencing depth. And we found that the variants closer to exons were more reliable, the variants in intronic regions flanking exons over ± 50 bps may be unreliable. This result suggests that we can pay more attention to variants closer to exons, while variants farther away from exons are more likely to be false variants, which need to be verified by Sanger sequencing.

The greatest number of deleterious variants was at the +5 and −5 positions, which was also the position at which many pathogenic variants had been reported in recent years [37–42], all above research found the rare +5 or −5 positions would cause exon skipping, splice site changing, splicing efficiency affected, and pathogenicity. As a result, many variants located at +5 and −5 positions caused kinds of rare genetic disorders. These suggest that +5 and −5 positions are potential pathogenic hot spot, we can pay more attention the ± 5 position when we analysis WES data. Especially when the patient has the typical genetic disease family history, and no pathogenic variants can be found in exonic region. When necessary, researcher can also do some functional verification experiments to confirm the harmfulness of the variant at the +5 and −5 positions. In our current study, we combined SPIDEX and dbscSNV to predict splice effects for more effective and comprehensive damaging analysis results. SPIDEX is a machine-learning technique trained on experimentally observed exon skipping events and predicts exon inclusion percentages based on genomic features, dbscSNV scores are two ensemble predictors of variant splice effects around canonical splice sites. They both had good performance in various performance evaluations [20, 21]. As SpliceAI (https://github.com/Illumina/SpliceAI) [43] can predict the effects of intronic variants on splicing, we will combine Splice AI to prove further statement in our follow-up study.

Through the analysis of statistical differences, we found that the number of unfiltered intronic variants was significantly different between most positions, but the number of intronic pass variants and deleterious variants was significantly different only in some regions, and there was no obvious pattern. The results of statistical differences analysis indicated that in the process of evolution the variation rate between different intron regions has non-significant difference. Because, while the variants that do not change the amino-acid sequence, such as intronic variants, are under lower evolutionary constraints, but for flanking intronic sequences, there was a higher level of conservation in mammals [44, 45]. Thus, there would not be a lot of variants in intronic regions flanking exons during evolution, the number of intronic pass variants and deleterious variants would not be significantly different only in most of the intronic regions flanking exons, and there was no obvious pattern, except for the special positions such as +2 and −2 positions, +5 and −5 positions, +9 and −9 positions.

## Conclusion

Our study revealed the characteristics of intronic variant in conventional WES testing data for the first time. We found that contrary to expectation, the number of intronic variants with QC passed was the lowest at the +2 and −2 positions but not at the +1 and −1 positions. The plausible explanation was that the former had the worst effect on trans-splicing, whereas the latter did not completely abolish splicing. And surprisingly, the number of intronic pass variants was the highest at the +9 and −9 positions, indicating a potential splicing site boundary. The FP in the intronic regions flanking exons generally accorded with “S”-shaped curve. At +5 and −5 positions, the number of variants predicted damaging by software was most which was also the position at which many pathogenic variants had been reported in recent years. And we found variants in intronic regions flanking exons over ± 50 bps might be unreliable.

This result can help researchers find more useful variants and demonstrate that WES data is valuable for intronic variants analysis. Although the 269 samples in our study is still not very sufficient, G*Power software [46] was used for calculating sample size and power for our statistical methods, about 54 samples can meet medium calling effects of t-test in our study, about 138 samples can meet medium calling effects of Fisher’s exact test. In the current study, we showed results of preliminary statistical analyses, as the sample size increases, more data will be added to improve the characteristics of intronic variations in WES data.

## Supplementary Information

**Additional file 1**. All Annotated Pass Variants Information.

**Additional file 2**. The Number of Raw/Pass/Deleterious Variants and The Number of Genes associated with these variants.

**Additional file 3**. The Number Distribution of The Raw Intronic Variants.

**Additional file 4**. The FP Distribution of The Raw Intronic Variants.

**Additional file 5**. The Number Distribution of The Pass Intronic Variants.

**Additional file 6**. The Number Distribution of The Deleterious Intronic Variants.

## Data Availability

The original contributions presented in the study are included in the article/Supplementary Material, further inquiries can be directed to the corresponding author.
